# Experiences of Transition to Secondary School in the United Kingdom for Children With Cleft Lip and/or Palate: A Qualitative Study

**DOI:** 10.5334/cie.33

**Published:** 2021-09-01

**Authors:** Rachael Faulkner, Sian Trenchard, Catherine Taylor, Craig D. Murray

**Affiliations:** 1 Wythenshawe Hospital, Manchester University NHS FT, GB; 2 Royal Manchester Children’s Hospital, Manchester University NHS FT, GB; 3 School of Health & Medicine, Lancaster University, GB

**Keywords:** cleft lip, cleft palate, CL/P, qualitative, psychological support, school transition

## Abstract

This study explored the secondary school transition experiences of children with cleft lip and/or palate (CL/P). Data were collected via semi-structured interviews and analysed using interpretative phenomenological analysis (IPA). Participants were recruited from a National Health Service (NHS) specialist cleft service covering a large geographical area in the United Kingdom. Six participants with CL/P (aged 11–12 years old) in their first 12 months following transition to secondary school were interviewed. Four themes describe participants’ transition experiences: (a) managing and valuing difference: the impact on self-worth and identity; (b) managing and valuing difference within the social context; (c) disclosure and the process of informing others about CL/P; and (d) developing positive peer relationships. Children with CL/P experience several psychosocial challenges during the transition to secondary school. Professionals involved with working with and supporting these children (and their families), such as psychologists, school nurses or wellbeing staff, child psychiatrists, social workers, mental health nurses and paediatricians, should attend to these issues when preparing for this transition in order to foster resilience and adjustment.

## Introduction

Cleft lip and/or palate (CL/P) is one of the most common types of congenital facial differences (Mossey et al., 2009; Murray, 2002) with a prevalence of 9.25 (UK) and 10.20 (US) per 10,000 births (IPDTOC Working Group, 2011). A CL/P is a gap or opening in the lip and/or mouth that occurs when areas of the face do not fully join during foetal development. Clefts are usually repaired surgically in early infancy; however, further procedures are often required as children grow. Additionally, children with CL/P typically experience a range of additional difficulties, including feeding and dental problems, otitis media with effusion (glue ear), recurrent ear infections, hearing and speech difficulties and/or visible facial differences (Shkoukani et al., 2013).

As children embark upon the transition to secondary school, they experience disruptions in peer relationships (Pratt & George, 2005; Zeedyk et al., 2003), and incidences of bullying can increase as students seek to establish status within social groups (Pellegrini & Long, 2002). Subsequently, negative effects on self-esteem and anxiety during the transition period are widely documented (Wigfield et al., 1991). The transition to secondary school, which typically takes place in the UK at age 11–12, can be particularly challenging for youth with craniofacial differences (CFD) such as CL/P, with increased potential for teasing and/or bullying (e.g., Tierney et al., 2015).

During the transition to secondary school, there is an increased desire to be accepted by one’s peers; indeed, one qualitative study found that young people with a range of CFD (including CL/P) developed appearance-related concerns around this time (Marshman et al., 2009). For example, “making a good impression” was important, with children often attempting to conceal their CFD or seek corrective treatments ahead of their transition to minimise the potential for negative peer interactions. Kapp-Simon and McGuire (1997) observed the social interactions of secondary school-age children with a visible CFD (including CL/P) alongside peers without CFD during school lunch breaks. They found that children with CFD were less likely to initiate social interactions, more likely to be on the periphery of group conversations, and less likely to receive positive peer responses.

The above studies point to particular issues that children CL/P may encounter when transitioning to secondary school and with which they, therefore, may need support. In the UK, where the current study took place, specialist cleft services operate on a regional basis to provide multidisciplinary input to children and their families to optimise feeding, growth, speech and language skills, and social and psychological wellbeing (NHS England, 2013). This multidisciplinary team approach promotes psychological wellbeing, aims to prevent mental health difficulties in children and their families, and identifies and provides support to those in need of psychological intervention (e.g., Lockhart, 2003).

According to UK cleft service specifications, psychological support should be available to all children with CL/P and their families prior to the child’s transition to secondary school (NHS England, 2013). However, specific recommendations regarding this support are not standardized, and subsequently regional variation exists (Rumsey & Harcourt, 2007). In addition, the evidence base on which to develop such recommendations is lacking, due to limited research on the secondary school transition experiences of children with CL/P.

To fill this gap in the literature, the present study explored the lived experiences of children with CL/P when transitioning to secondary school in order to illuminate how they experience and make sense of this critical phase in their lives. Specifically, the purpose of the study was to inform the development of cleft and related services as well as the practice of psychologists and other health professionals working with children with CL/P.

## Method

### Sampling, Recruitment and Participants

Studies using interpretative phenomenological analysis (IPA) focus on small, homogenous samples (typically 4–10) to balance the time-intensive depth of detail and interpretation required for the idiographic analysis of cases (Smith et al., 2009). To help ensure homogeneity (that is, a common set of experiential features of research concern), children were eligible to take part if they were born with a CL/P and had transitioned from a mainstream primary school (covering ages 5–11) to a mainstream secondary school (ages 11–16) within the UK, in the previous 12 months.

Recruitment took place via a UK-based regional cleft service. The cleft team identified and contacted the parents/carers of all individuals who met the inclusion criteria with information about the study. Families were contacted in batches until the target sample size was reached. The obtained sample comprised six participants (1 female, 5 male). Brief biographical details are provided in ***Table 1***.

**Table 1:** Participant Biographical Details.


JOSH

Josh, 12 years old, was born with a cleft palate. He perceived that he sounded different from peers because of his cleft palate; however, speech difficulties were not overly apparent to the researcher during the interview. Josh described himself as very confident and well-liked by peers. Josh chose to attend a different secondary school from his older sibling so that he could attend the same school as several pre-existing friends. He also had two younger siblings.

**ETHAN**

Ethan, 11 years old, was born with visible cleft lip and palate (which affected his speech) and additional congenital health difficulties, including renal problems. Ethan reported two significant episodes of bullying (one at primary school, one at secondary school). Ethan had one younger sibling (without a CL/P).

**HARRY**

Harry, 11 years old, was born with a visible cleft lip and palate, which affected his speech. Harry’s family relocated shortly prior to his transition to secondary school; therefore, he started secondary school without any of his peers from primary school.

**GEORGE**

George, 11 years old, was born with a sub-mucous cleft palate. He described having speech difficulties that much improved following surgery and speech and language therapy. George perceived his cleft palate to be largely invisible to others, except when attempting to eat/drink certain foods (e.g., ice cream and milkshakes). George had an older sister (without a CL/P), who attended the same secondary school as him. As part of the school transition process, George and his school peers read Wonder, a children’s novel about visible facial difference by Raquel Jaramillo (Palacio, 2012).

**RUBY**

Ruby, aged 11, was the only female in the sample. Ruby had a visible cleft lip and palate and described feeling self-conscious about her appearance as a result. Ruby described several experiences of appearance-related bullying throughout primary and secondary school. She reported receiving previous psychological input from her local cleft service for pre-operative anxiety, which she described as beneficial. Ruby had two younger brothers (without a CL/P).

**TYLER**

Tyler, aged 12, was born with a visible cleft lip and palate. In preparing to transition to secondary school, Tyler attended a mainstream “summer camp” at his new secondary school. In addition, he also attended a school transition preparation group for people with CL/P run by his local cleft psychology service. At the point of interview, Tyler had been at his secondary school for seven months.


### Data Collection

The National Research Ethics Service (NRES) granted approval for the study, and fully informed written parental consent and participant assent was provided prior to participation. Data were collected via a single face-to-face interview with each participant lasting an average of 48 minutes (range 41–63 minutes). All interviews took place at the participants’ homes, and all opted to have their parent/carer present.

A semi-structured approach was utilised, in which the first author facilitated an exploratory discussion with participants about their experiences of transition to secondary school. An interview schedule comprised of broad open-ended questions related to the primary research question was developed following consultation with a health psychologist with expertise in qualitative research (fourth author) and a clinical psychologist working with the study population (second author) and was used flexibly to guide interviewing. Examples of questions included “What has it been like growing up with a cleft?” and “Can you tell me what it was like starting your new school?” Followup questions, such as “Can you tell me more about that?,” prompted participants to expand on their accounts. The interview schedule facilitated a consistent approach to questioning, while allowing exploration of nuances in participants’ experiences in a conversational manner.

### Data Analysis

A qualitative design was chosen to allow for the indepth, idiographic examination of participants’ experiences within an exploratory framework. Interpretative phenomenological analysis (IPA; Smith et al., 2009) was selected as the most appropriate methodological approach to address the research aims due to its underlying philosophical principles rooted in phenomenology, hermeneutics, and idiography. *Phenomenology* involves understanding the meaning of lived experience; *hermeneutics* encompasses the interpretation of bodies of text, such as research interview transcripts; and *idiography* concerns the sense an individual makes about their lived experience and the essence of what this experience is like for them. IPA, therefore, focuses on the lived experiences of individual participants alongside any recurring experiences for a particular, well-defined group (Smith et al., 2009).

Data were analysed using IPA, following guidelines by Smith et al. (2009). Specifically, the first author read each transcript a number of times to aid familiarisation, and then began coding the data. This involved annotating the left margin of each transcript with exploratory comments that highlighted anything of interest to the research question from a psychological perspective. For instance, this included a summary of the thoughts, feelings, and actions shared by the participant and any preliminary interpretations about phenomena (Smith et al., 2009). Next, the right margin was used to summarise the fundamental essence of the data in the form of key words and phrases that constituted emergent themes. *Emergent themes* reflect an initial understanding about the participant’s experience taking into account both the first-order participant words and the second-order author interpretations (Smith et al., 2009). Emergent themes were grouped into a coherent structure across each participant’s account as a whole. Narrative descriptions were then written for each theme to provide a coherent, indepth summary.

Next, themes from each participant were collated and reviewed so that patterns across the sample could be examined (Smith et al., 2009). This included a focus on both similarities and divergences, within and across the data. This part of the data analysis was carried out in conjunction with the fourth author, who examined the audit trail of the analysis and contributed to the titling and interpretations of themes. Related themes were combined and modified theme titles assigned to capture the essence of each overarching theme (Smith et al., 2009). The final stage of analysis involved developing saturated narratives of each overarching theme encompassing the data in their entirety. These accounts will be presented as the results, and evidenced by extracts from between four and six participants for each theme, exceeding Smith’s (2011) “acceptable” criteria for reporting IPA studies with a sample size of four to eight participants.

### Reflexivity

In IPA, research findings are co-constructed via the interface between the researcher and the participant. Reflexive practice is essential to enable researchers to acknowledge the impact that their own assumptions may have on findings. Smith et al. (2009) provide a framework for data analysis that encourages researchers to identify and then temporarily set aside their preconceptions in order to privilege participant accounts. Accordingly, an audio diary was used throughout the research process alongside a reflective log to enable consideration of the process and content of data collection and analysis and aid reflexivity.

## Results

Four overarching themes describe participants’ experiences of transition to secondary school: (a) managing and valuing difference: the impact on self-worth and identity; (b) managing and valuing difference within the social context; (c) disclosure and the process of informing others; and (d) developing positive peer relationships. In the following, we present each of these themes evidenced by data excerpts from participants (all of which are anonymised and use pseudonyms).

### Managing and Valuing Difference: The Impact on Self-Worth and Identity

Theme 1 describes participants’ sense of feeling different from their peers as a result of their CL/P, the impact that this has had on their self-worth and identity, and how they managed this during the transition to secondary school.

All participants thought that their CL/P meant that they were in some way different from peers; for example, having a different appearance (Ruby and Tyler), sounding different (Josh and George), or a general sense of being different (Ethan and Harry). This was due to the rarity of CL/P, as most stated they did not know anyone else with CL/P at either their primary or secondary school.

The transition to secondary school magnified participants’ sense of difference and brought “difference” into conscious reflection. Some considered difference to be special and/or something to feel proud of; “*I look different and I had braces [before anyone else did] … I felt different but not in a bad way, I was kind of proud”* (Ruby; 169–178). Such views positively influenced self-worth. However, feeling different was also paradoxically experienced as emotionally challenging, with feelings of discomfort, embarrassment, and isolation being common; “*No one knows what it’s like, and no one knows what it feels like and it’s a bit like I wish someone, just one person knew what it felt like”* (George; 351–352).

Negative emotional reactions were driven by an underlying sense of undesirability and a belief in the “truth” of this undesirability; *“This boy came up to me … for no reason he called me fat nose … I was really embarrassed … the fact that he kind of, I can’t explain it, like, the fact that it was kind of almost true”* (Ruby; 4–15). However, because most participants both positively and negatively appraised their CL/P and sense of difference, the impact that negative social interactions had on self-worth was complex. To highlight this, on the one hand, Josh indicated that he considered his difference to be special and interesting to others; “*I knew people were happy about me and like they were fascinated by me, by my cleft palate, cause like it’s not like a great population of people with them”* (Josh; 339–340). But on the other hand, feeling different from his peers also resulted in feelings of inadequacy. For instance, Josh often used language laden with negative connotations such as *“dodgy”* and *“hole mouth”* to describe his CL/P and experiences of difference during his transition. In viewing his cleft as integral to his identity, Josh felt more threatened by negative stereotypes (real or perceived) surrounding his difference. These contradictory and complex patterns appeared across most participants. Subsequently, it seemed that feeling “different” was neither a wholly positive nor a wholly negative experience for participants and could be both: *“Sometimes it’s good being different, sometimes I don’t mind it that much, and sometimes I like being different”* (Ruby; 459–460).

Participants attempted to cope with feelings of difference during their transition by highlighting shared interests and experiences with peers without CL/P, to enable them to feel “Less different.” In doing so, some participants also began to notice the wide range of individual differences that exists in others. Thus, understanding the “normality of difference” encouraged a positive view of difference, which in turn fostered a positive sense of self. As Tyler explained:

*I’m different from other people and I don’t mind that … it can be a bit of appearance, and personality …, like my nose is a bit different I know that, and I think my personality is [different]. Well, everyone’s personality is kind of different … It’s a good thing, because if we were all the same, we would get tired of it … same personality and everything, and then everyone would just go crazy and it would be horrible*. (402–411)

A further strategy adopted by two participants (Ruby and Ethan), particularly when faced with external threats such as bullying, was to seek validation through reflecting on their own positive attributes. Positive attributes focused on participants’ constructions of how they considered themselves a “good person,” as opposed to physical characteristics. For example, Ruby reflected on some charity fundraising that she participated in and stated, *“I had done something to help others and it made me feel a bit better about myself… the fact tha t… it didn’t matter how I looked, just that I had done something good for other people”* (73–79).

Overall, participants felt “different” from their peers due to their CL/P. However, difference was both positive and negative for them and, therefore, both positively and negatively influenced their sense of self-worth. Nonetheless, participants adopted a range of strategies to enable them to manage feelings of difference during the transition to secondary school.

### Managing and Valuing Difference Within the Social Context

Theme 2 describes the ways in which participants managed their “difference” within the social context during the transition period to facilitate a sense of social acceptance.

As they embarked upon their transition to secondary school, participants worried about the impact that their CL/P would have on their social experiences. Most of them were apprehensive about negative appraisal, and concerns regarding bullying were apparent, especially among participants with previous experiences of teasing/bullying;

*I was sort of a bit worried if I would get bullied about it … I didn’t want people to know and then pick on me because I had that [CL/P], like in case they called me names or I was different and they might make fun of that”* (George; 81–85).

Participants adopted a range of strategies to manage their sense of difference from peers during their transition. Some sought to minimise or hide their “difference” to limit negative peer appraisal. For instance, Josh explained how he initially avoided shouting during sports lessons at his new school to try to prevent others from noticing he sounded different; *“I didn’t like shouting in rugby. I didn’t want to make my voice [obvious to others] so that people ask what’s wrong with my voice”* (Josh; 308–309). However, hiding one’s difference contradicted beliefs that difference can be positive. For instance, in addition to hiding his difference, Josh also described using his cleft palate as a means of impressing peers; *“I show them [the scars in] my mouth… they are all impressed”* (Josh; 13–17). This again highlights the complex relationship that participants had with a sense of difference, both personally (as described in Theme 1) and socially.

Hiding/minimising CL/P from peers was not an option for participants such as Ruby and Ethan, who had a more visible CL/P, and both described experiencing appearance- related teasing/bullying shortly after starting secondary school. Teasing/bullying during the transition period was experienced as emotionally difficult as it threatened participants’ sense of feeling accepted in their new environment. As Ethan shared:

*Two girls in my [class] were making fun of me because of my appearance … it made me angry and upset … I was shocked because I didn’t really think that people would be like that [at secondary school]. When I looked around the school, a lot of people were being nice to each other”* (139–155).

To cope with this, these students avoided being the centre of attention, preferring smaller peer groups that would afford a sense of safety. Having good support when issues arose was also important. For instance, both Ruby and Ethan stated that having supportive parents and teachers meant that they felt able to report the bullying and seek the help they needed;

*I went out and told the teacher, and they told them off quite badly … it made me cry [to repeat it to the teacher] but I think it helped … I thought that if she [the other student] wasn’t [going to] get into trouble then she was going to do it again* (Ruby; 36–40).

Participants also sought a sense of “social normalisation” to reduce the threat of feeling different during the transition period. For instance, George described how he was just the same as his peers without CL/P because he could do all the same things as they could do. Alternatively, Josh described how he would attempt to fit in with peers by reciprocating teasing regarding physical characteristics to normalise and trivialise the teasing he received and be seen by others as “the same.” For instance, *“I’d call them and they’d call me … I’d call them peg leg or something, so we’d just call each other’s illnesses”* (Josh; 48–50). Additionally, Tyler found that he was able to normalise his experiences of difference by meeting other peers with a CL/P at a specialist CL/P school transition group. *“I remember going to the school [transition group] thing at the hospital, and I met a few people … it was good, seeing other kids with a cleft lip … it was just nice to see other people like that”* (Tyler; 276–291).

Feeling the same as others reduced feelings of isolation and increased a sense of connectivity, which participants linked to adjusting and coping during their transition.

In summary, participants worried that “difference” might affect their ability to fit in and feel accepted by peers. Active attempts to seek a sense of “normalisation,” establish connections with peers, and gain support from others facilitated adjustment and coping despite initial anxieties.

### Disclosure and the Process of Informing Others About CL/P

As Theme 3, participants described the process of informing others about CL/P during transition to secondary school, and the different ways in which they managed disclosure.

As they started secondary school, participants noticed that new peers and teachers had little understanding of CL/P, and in some cases this led to incorrect assumptions being made; for example, “someone once asked me if it was a car crash, and I was like NO!” (Ruby; 220). The novelty of CL/P invoked curiosity, and most participants reported being asked questions about their CL/P. Most participants interpreted this curiosity as a genuine, friendly attempt by peers to understand more about CL/P. As a result, they orientated towards informing others about CL/P to increase understanding and put others at ease. However, not all agreed. Ruby and George both considered CL/P to be a private matter and, therefore, experienced questioning as invasive; *“*I kind of wanted it to be private, …. I just didn’t want everyone knowing … because like, I think it might have given me another reason to be singled out” (Ruby; 253–260). Accordingly, Ruby and George sought to maintain their privacy; however, this was often difficult, given the visibility of their CL/P. Subsequently, Ruby tended to ignore or divert attention away from her CL/P to enable her to retain some control in disclosure; “I tried to ignore them I was like ‘pardon what did you say?’ and then tried to think of a way to avoid answering it … sometimes I just shrugged and walked off” (Ruby; 184–207).

Sharing with peers was largely spontaneous and in response to peer questioning; *“I told them because they asked me”* (Tyler, 40). Participants took a matter-of-fact approach, describing in literal terms what having a CL/P means; “I say I was born with it; I was just born with a hole” (Harry; 286). This approach was influenced by discussions with healthcare professionals during clinic visits and broader family discourses; “I just explained everything that my mum and dad had told me and that I had heard from the doctors” (Ethan; 65–66). Having support from pre-existing friends was also beneficial when explaining to new peers. Josh described how pre-existing friends understood enough information to explain CL/P to others, whilst lacking an emotional connection that might make talking about CL/P difficult.

My friends told my new friends … they explained it probably better than me … they put more detail, like about my operations, and how much pain I was in … and what age I was when I had it … cause like I was a bit nervous and they wasn’t [sic]because it’s not them (Josh; 173–183).

However, participants also reported experiencing difficulties informing others. Most felt unprepared to talk about their CL/P, due to having little prior experience of informing others. They also highlighted that they themselves did not have a comprehensive understanding of CL/P, which resulted in feelings of inferiority for not knowing better. For instance, George explained, “I didn’t know what myself had, so like I felt a bit silly … like because I was born with it … and I didn’t even understand it, I felt a bit silly” (68–74).

However, participants became more confident in talking about their CL/P as they gained more practice in doing so; “I done it once, it felt ok to do it again” (Josh; 185). Positive reactions from others also boosted participants’ confidence in informing others; *“*I opened up and sort of told someone and it made me feel better to think that they weren’t doing anything about it” (George; 214–216). Several participants also described practicing potential responses to peer questioning ahead of their transition to increase a sense of preparedness. For instance, George described how he got the idea of practicing comebacks to unwanted peer questioning after reading Wonder, a children’s novel about visible facial difference by Palacio (2012).

Finally, the decision to disclose was not always straightforward, with participants indicating ambivalence regarding how much they shared and with whom. Timing was also an important consideration. Josh described a need to inform others (especially teachers) early on to alleviate misunderstandings; “If I tell [my teacher early on about how CL/P affects my voice,] then he won’t pull me up on it in the future” (Josh; 192). Conversely, both George and Ruby only shared their experiences with close friends several weeks/months into the new school year once positive peer relationships had been established; “I made friends and then I trusted them so I thought I could tell them … I thought if they really are my friends they wouldn’t really laugh or anything” (George; 89–92).

In summary, participants experienced curiosity and questioning from peers, and most felt unprepared to respond. Participants’ management of disclosure varied markedly. Nonetheless, a focus on increasing preparedness reduced anxieties regarding disclosure.

### Developing Positive Peer Relationships

Theme 4 illuminates the influence that CL/P had on participants’ experiences of developing positive peer relationships during the transition period and the ways in which they negotiated the challenges they were facing.

All participants started secondary school with some, but not all, of their peers from primary school except Harry, whose family relocated shortly before the transition. Developing positive peer relationships and feeling liked and accepted by new peers was a pivotal aspect of participants’ transitions. Participants worried about the impact that their CL/P would have on their ability to make friends and feel included; *“*The thing is, the fact I look different, people might judge me on the way I look, not the way I am because they don’t want to know me because I look different” (Ruby; 482–484). Nevertheless, meeting new peers was also an exciting prospect, and most described looking forward to opportunities to make new friends and feeling optimistic about their chances of peer success; *“I was hoping I was going to make friends”* (Harry; 110).

A key driver behind participants’ emphasis on developing positive peer relationships was ensuring access to peer support to reduce a sense of vulnerability; *“… I*f you are with people you don’t feel as vulnerable, even if they are not strong or anything you still don’t feel as vulnerable as when you are alone …” (Tyler; 431–432).

Participants made use of a number of resources and strategies to assist in developing positive peer relationships. Maintaining links with old friends whilst building new relationships seemed to help foster a sense of familiarity and connection. Having a few close friends from primary school transitioning to the same secondary school and/or making a few new friends early on in the transition process was also helpful. Participants utilised these existing friendships as a safe base through which to further extend peer networks. As Harry explained; *“Having friends who are friends with other people and having things in common then meeting them”* (215–216). Older peers were a key resource when navigating the challenges of starting secondary school. Older peers included school organised “buddies” (Ethan) and informally developed acquaintances such as older siblings of friends (Josh).

Further strategies included emphasising commonalities with peers with a focus on shared interests to avoid and/or minimise any observable CL/P-related differences and attempting to be seen as “nice” and likable, as described by Tyler; *“If you are friendly to people you get more friends … it’s just being friendly is nice”* (97–98). Making a good impression early on in the transition process was also important to participants, as was portraying confidence and self-acceptance; *“I make new friends every day, every step I take … lots of people follow me … because I act all confident”* (Josh; 303–306), and *“Because I accept it [CL/P] as part of me so they do the same of me”* (Josh; 21–22).

However, whilst this belief was empowering to some participants, it seemed to be underpinned by a sense of non-acceptance from others (as indicated by the need to hide/minimise CL/P from others highlighted in Theme 2). Accordingly, a number of participants attempted to pre-select new friends according to perceived friendliness as a way of avoiding potential bullies who may threaten a sense of acceptance from others. As Tyler explained, *“There has been a few [peers] that I have not bothered going near because they look like they would be mean … you don’t want to go near them ‘cause you feel like they will be a bit hostile …”* (236–240). However, choosing the “correct” friends required a judgment based on perceived, rather than actual, characteristics, which contradicted participants’ own desires to not be taken at face value.

Developing positive peer relationships required a significant amount of effort. Despite this, all participants described how they felt their efforts had paid off as they had established a number of true friendships with new peers. “True friends” were those that participants felt a sense of shared unconditional acceptance and respect with; *“I do think about [being different] quite a lot but when I am with my friends I don’t really think about it that much because they don’t really make fun of me, otherwise I wouldn’t be their friend”* (Ruby; 477–480). Support from true friends was considered to be reciprocal, with participants emphasising that they too provided support and encouragement to peers (without CL/P) highlighting that the need for peer support goes beyond CL/P related issues. Developing positive peer relationships was a pivotal, yet challenging aspect of participants’ transition experiences. Nonetheless, they were able to adopt a range of strategies to enable them to establish new and successful friendships.

## Discussion

This research aimed to understand the lived experiences of children with CL/P when transitioning from primary to secondary education. The resulting analysis highlights the range of psychological and social challenges that participants faced; specifically, the impact that feeling “different” had on participants’ sense of identity and self-worth, and on their social interactions with peers during the transition period. Findings also highlight the many ways in which participants adapted to and coped with these challenges.

Underpinning the first two themes was the notion of “difference.” As with previous studies, participants expressed a sense of being different from peers because of their CL/P (Chapados, 2000; Chetpakdeechit et al., 2009). However, findings from the current study also highlight that participants’ personal relationship with “difference” was complex and, at times, contradictory. The transition to secondary school magnified feelings of difference, and concerns regarding fitting in and being accepted increased. However, as found in a study by Egan et al. (2011) that focussed on the experiences of people who identify themselves as having adjusted positively to a visible difference, the use of active coping strategies (such as talking about CL/P to increase understanding in others, accessing support from parents, teachers, and peers, and maintaining a focus on one’s own positive attributes) facilitated positive adjustment.

Developing positive peer relationships was important to participants during the transition and was seen as a way to reduce a sense of vulnerability and increase feelings of resilience and acceptance. This is consistent with the existing literature, which suggests that having a close friend may offer protection against teasing and bullying (Acquah et al., 2016; Division of Clinical Psychology, 2010; Hodges et al., 1999).

However, findings from Themes 3 and 4 highlight the many social challenges that participants had to negotiate when developing positive peer relationships. A widespread lack of awareness and understanding of CL/P was apparent in participants’ descriptions of interactions with peers (and, in some cases, teachers). Unsolicited curiosity and questioning about their CL/P were commonplace, and participants felt unprepared for how to respond to this attention. Experiences of teasing and bullying were also highlighted by a number of participants, mirroring existing research evidence (e.g., Chapados, 2000). However, unlike previous studies, participants largely felt supported by teachers when bullying occurred. In addition, whilst bullying was consistently considered to be a painful emotional experience, teasing could be seen to strengthen peer relationships depending on the individual’s perception of it. Therefore, the attributions that an individual makes about the challenges they face during the transition period and their sense of preparedness and confidence in dealing with these challenges are likely to influence adjustment.

### Service Implications

Targeted specialist transition programmes for children with CL/P (Maddern et al., 2006; NHS England, 2013) play a key role in increasing students’ preparedness for the transition experience. However, only one participant from this study attended such a programme. Whilst participation in such programmes was not a focus of this study, findings may nonetheless illuminate barriers to attendance.

First, participants broadly felt that they coped well during the transition period despite apparent challenges. Second, as highlighted in Themes 1 and 2, participants had a complex relationship with “difference,” and at various points during the transition period they distanced themselves from their “difference” in an effort to feel more confident in their ability to fit in and establish positive peer relationships. The act of distancing may explain why some participants chose not to engage in specialist support at this time.

In addition to existing programmes, cleft services should consider alternative ways of supporting children with CL/P through the transition to secondary school in order to reach a wider audience. This could include a greater emphasis on the role of parents in supporting children at home to increase preparedness; for example, practicing responses to peer questioning and/or supporting parents to share information about their child’s CL/P with the new school to increase understanding amongst teachers. The development of age-appropriate mobile phone/tablet-based apps incorporating material from specialist cleft transition programmes may also help normalise transition preparation by removing it from a hospital-based environment (which may reinforce a sense of difference) and placing it into an accessible format that children can access at their own pace.

A focus on increasing understanding of CL/P in the general school population, and normalising difference more broadly, could also help further improve transition experiences of children with CL/P. Indeed, evidence suggests that children respond well to school-based programmes aimed at raising awareness of appearance-related issues (Lovegrove & Rumsey, 2005). Within the UK, most schools already implement general transition programmes for all pupils starting secondary school (Evangelou et al., 2008). One participant in this study discussed how as part of this general transition preparation, students in his school read the children’s novel *Wonder* about a boy who was born with Treacher Collins syndrome and a cleft palate. Whilst this initially increased curiosity from peers, feelings of preparedness, acceptance, and normalisation also increased. Psychologists working in cleft and/or other paediatric services could, therefore, work with local schools to develop more inclusive transition programmes that have a broader focus on normalising differences and supporting inclusivity for all pupils.

### Limitations

Participants in this study were self-selecting (via parent gatekeepers). It is, therefore, possible that only those individuals who felt they had coped well with the transition to secondary school opted to take part. Potential ways in which to have addressed this issue, and thereby strengthen the analysis, would have been to include a stage where others who have CL/P could comment on the emerging analysis with a focus on whether the findings resonated with them and then have the original participants verify the analysis of their data.

A further limitation concerns the generalisability of qualitative research to other contexts and demographic groups (Myers, 2000). In particular, with the exception of one participant, all children were males. This may mean that data collection and analysis was not sensitive to particular gendered experiences and approaches to managing transition. Nonetheless, this study is the first to examine the lived experiences of children when transitioning to secondary school and provides useful service implications and directions for further research. The accumulation of similar studies over time may allow for further generalisations of the data (Smith et al., 2009).

### Directions for Future Research

This study highlights several areas requiring further research. First, research utilising longitudinal designs that follow participants through the transition process could enhance findings from the present study and further explore the impact of transition on secondary school in children with CL/P. Specifically, research exploring the underlying processes of resilience at this time period is recommended. In addition, exploration of the efficacy of specific transition preparation programmes (both specialist CL/P and general school-based) would also be advantageous.

## Conclusions

This novel exploratory study provides insight into the lived experiences of children with CL/P when transitioning to secondary school in the UK. Findings highlight that issues regarding difference, as well as peer reactions and relationships, pose challenges for children with CL/P during this time. Findings also highlight the range of coping strategies that enabled participants to develop resilience during the critical school transition period. Professionals working with and supporting children with CL/P (and their families), such as psychologists, school nurses or wellbeing staff, child psychiatrists, social workers, mental health nurses, and paediatricians, should attend to these factors when supporting young people with CL/P (and their families) in preparing for the transition to secondary school and consider broader ways of further enhancing resilience and coping.
